# Biogenic Phosphonate Utilization by Globally Distributed Diatom *Thalassiosira pseudonana*

**DOI:** 10.3390/microorganisms12040761

**Published:** 2024-04-10

**Authors:** Huilin Shu, Yuan Shen, Hongwei Wang, Xueqiong Sun, Jian Ma, Xin Lin

**Affiliations:** 1 State Key Laboratory of Marine Environmental Science, Xiamen University, Xiamen 361005, China; shuhuilin@jxnu.edu.cn (H.S.); yuanshen@xmu.edu.cn (Y.S.); wanghongwei@stu.xmu.edu.cn (H.W.); 22320220156409@stu.xmu.edu.cn (X.S.); jma@xmu.edu.cn (J.M.); 2 College of the Environment and Ecology, Xiamen University, Xiamen 361005, China; 3 College of Ocean and Earth Sciences, Xiamen University, Xiamen 361005, China; 4 College of Life Sciences, Jiangxi Normal University, Nanchang 330022, China

**Keywords:** phosphonate, bioavailability, diatom, 2-AEP, 2-AP3

## Abstract

Phosphonates are a class of organic phosphorus (P) compounds that contribute ~25% of dissolved organic P. Recent studies reveal the important role of phosphonates mediated by prokaryotes in the marine P redox cycle. However, its bioavailability by eukaryotic phytoplankton is under debate. 2-Aminoethylphosphonic acid (2-AEP) and 2-amino-3-phosphonopropionic acid (2-AP3) are two biogenic phosphonates in the marine environment. Here, *Thalassiosira pseudonana*, a common diatom species in the ocean, is able to recover growth from P starvation when provided with 2-AEP and 2-AP3. Moreover, 2-AEP cultures exhibited a more similar growth rate at 12 °C than at 25 °C when compared with inorganic P cultures. The cellular stoichiometry of 2-AEP groups was further determined, the values of which are in-between the P-depleted and DIP-replete cultures. This study provides evidence that biogenic phosphonates could be adopted as alternative P sources to support diatom growth and may provide physiological adaptation.

## 1. Introduction

Phosphorus (P) is an essential nutrient for the growth of living organisms. Dissolved inorganic P (DIP), the preferred form of P used by phytoplankton, is often a limiting nutrient for phytoplankton in marine environments [1,2,3]. Thus, dissolved organic P (DOP) has emerged as a prominent alternative P source [4,5].

Phosphonates are a class of organic P with a chemically stable C-P bond. Besides synthetic compounds (e.g., herbicide glyphosate), biogenic phosphonates produced by various organisms are present in the ocean [6,7]. Two biogenic phosphonate compounds, 2-aminoethylphosphonate (2-AEP) and its derivative, 2-amino-3-phosphonopropionic acid (2-AP3), are the composition of membrane phospholipids in many organisms, such as prokaryotes and mollusks [8,9]. A recent study shows that *Prochlorococcus* likely allocates over 40% of cellular P towards phosphonate production in the ocean [10]. Genome surveys suggest that de novo synthesis of 2-AEP is performed in corals [11]. Therefore, exploring the bioavailability of biogenic phosphonates by phytoplankton has considerable importance. 

Compared with the well-elucidated metabolism of phosphonates in prokaryotes [12,13,14], discrepancies were found in a few studies of eukaryotic phytoplankton [15,16]. Picoprasinophyte *Micromonas commode* and coccolithophore *Emiliania huxleyi* are able to utilize 2-AEP, whereas diatom *Phaeodactylum tricornutum* failed [17]. However, our recent study showed that P-starvation-treated *P. tricornutum* can recover growth with a 2-AEP supplement, and the utilization is mediated by endocytosis and integration into membrane phospholipids (DAG-2-AEP, diacylglyceryl-2-AEP) [16]. Furthermore, an in silico analysis of the global meta-omic atlas suggests that the associated utilization functional genes are prevalent in diatom assemblages and actively expressed in the cold regions [16]. 

On these grounds, a hypothesis that the cosmopolitan diatom *Thalassiosira pseudonana* [18,19,20] is able to utilize biogenic phosphonates and the metabolic activity is temperature-sensitive is proposed. Here, the bioavailability of 2-AEP and 2-AP3, which share similar chemical structures and are components of membrane phospholipids, was investigated [8,9]. Then, the physiological responses of algae grown with phosphonate supplements were examined at different temperatures. 

## 2. Materials and Methods

### 2.1. Cell Culture and Experiment Setup

*T. pseudonana* was provided by the Center for Collections of Marine Bacteria and Phytoplankton of Xiamen University, China. Two batch experiments were conducted to (batch 1) examine the bioavailability of 2-AEP and 2-AP3 (Sigma-Aldrich, St. Louis, MO, USA) and (batch 2) explore the physiological response under the conditions of significant temperature differences (Table 1). As documented in other studies, 12 °C and 25 °C (comparable to those of 20 °C) represent the temperatures with the lowest growth rate and the highest growth rate, respectively [21]. Before the experiments, seed cultures were subject to P starvation for 8~10 days until the ambient phosphate concentration was below ~0.3 μM and the cell growth ceased. Antibiotics were applied to inhibit the growth of bacteria (Table 1).

### 2.2. Determination of Cell Density and Fv/Fm

Cell density was measured daily by using a CytoFLEX flow cytometer (Beckman Coulter, Brea, CA, USA) and estimated by gating areas in the chlorophyll A versus SSC-A dot plot generated from a 1 mL cell sample. Fv/Fm was determined using a FIRe Fluorometer System (Satlantic, Halifax, NS, Canada). Prior to the measurement, 1 mL of the cell sample was subject to dark adaption for 20 min and then processed following the manufacturer’s protocol [22].

### 2.3. Cellular C and N Content

Cells were collected using pretreated GF/F membranes. The collected cells were dried at 60 °C for 8 h. Then, 500 μL of 1% HCl was dripped onto the filters, and the filters were dried at 60 °C for 12 h again. After the cells were pretreated [16], the cellular C and N contents were determined using a Vario EL cube analyzer (Elementar Analysensysteme GmbH, Langenselbold, Germany) in accordance with the reported method [23]. 

### 2.4. Determination of DIP and P Content

Cells were filtered onto GF/F membranes and resuspended in 25 mL of distilled water. The suspension was digested by adding 4 mL of 50 g/L potassium persulfate and autoclaving at 121 °C for 30 min [24]. Then, the cellular P content of the digested suspension and the DIP concentration of the filtrate were determined using the molybdenum method [24].

### 2.5. Statistical Analysis

Differences among different P and temperature treatment groups were measured with *t*-tests.

## 3. Results and Discussion

### 3.1. Differential Growth-Promoting Effects between 2-AEP and 2-AP3

**Figure 1:**
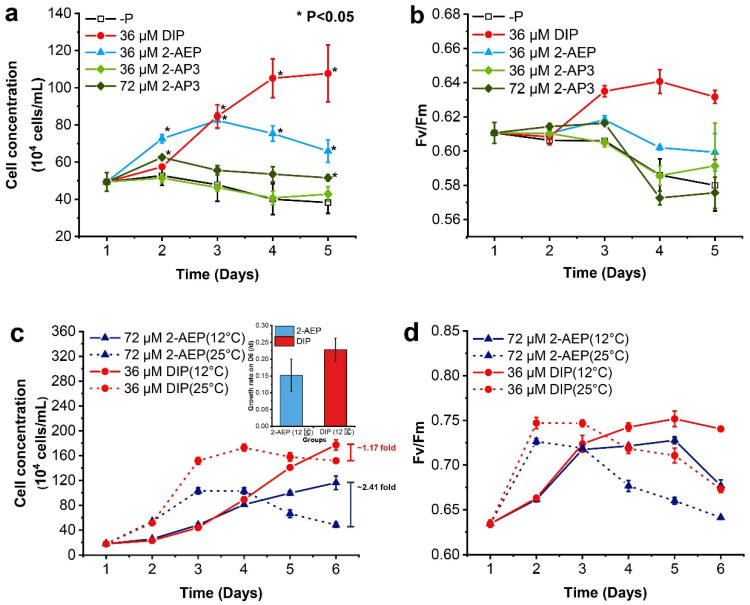
Physiological responses of *T. pseudonana* to different phosphorus levels ((**a**), growth curve; (**b**), Fv/Fm) and temperatures ((**c**), growth curve; (**d**), Fv/Fm). The culture temperature was 20 °C (**a**,**b**) and 12/25 °C (**c**,**d**). Each culture group was set up in biological triplicate. The error bar represents the standard deviation of the mean values. The inner panel of (**c**) represents the growth rate on D6 at 12 °C, and * represents a significant difference (*p* < 0.05) between P-depleted and other groups.

Different growth-promoting effects were observed in the batch 1 culture. After P starvation, *T. pseudonana* was able to recover growth significantly in the medium supplied with 2-AEP (36 μM) and 2-AP3 (72 μM), respectively (*p* < 0.05), while failing to grow in the 2-AP3 supplement (36 μM, Figure 1a). The cells in the 2-AEP (36 μM) group continued growth and peaked at the maximum cell concentration of 8.24 × 10^5^ cells mL^−1^ at Day 3, then declined gradually, and Fv/Fm exhibited a similar pattern accordingly (Figure 1a,b). In comparison, we observe mild growth promotion in the 2-AP3 group. After the supplement of 72 μM 2-AP3, instant cell growth was recovered in 24 h, showing a growth rate of 0.24 μ d^−1^, which is about half of that in the 2-AEP (36 μM) group (Figure 1a). After then, cells ceased growth and declined towards the end of this experiment, accompanied by an abrupt decline in Fv/Fm (Figure 1b). Maximum cell density observed on D2 was 6.26 × 10^5^ cells mL^−1^, about half of that in the 2-AEP (36 μM) group. 

DIP was barely detected (lower than the detection limit; ~0.3 mM) in the 2-AEP and 2-AP3 groups, demonstrating that 2-AEP and 2-AP3 can be utilized as an alternative P source by *T. pseudonana*. Though provided in the same or higher concentration, lower cell density acquired in both phosphonate groups suggests limited utilization efficiency compared with DIP, which is common according to previous reports [15,17]. Furthermore, lower Fv/Fm indicates repressed photosynthesis, suggesting that *T. pseudonana* cells were under P stress in both phosphonate groups.

2-AP3 is the derivative of 2-AEP, known as a component of phospholipids in cell membranes. Studies have demonstrated that 2-AEP and its derivatives can be incorporated into phospholipids in cell membranes [25,26,27]. 2-AP3 can be decomposed via a transamination reaction and decarboxylation to 2-AEP in *Tetrahymena* [28]. Given the limited utilization of 2-AP3 observed in the present study, the other possible metabolic pathways cannot be completely excluded. The underlying mechanism of 2-AP3 utilization by *T. pseudonana* and its potential bioavailability need to be further investigated to address the knowledge gap in eukaryotic phytoplankton.

### 3.2. Different Growth Strategies under Variable P Nutrients and Temperatures

A recent study of the global ocean gene atlas shows an enriched distribution of representative genes of the proposed 2-AEP utilization mechanism by diatoms in low-temperature waters [16]. Therefore, the batch 2 experiment was conducted to further explore the cellular physiological response grown with different P nutrients and temperatures, in which 72 μM of 2-AEP was provided to obtain more significant differences for the comparative analysis.

Different growth patterns that are temperature-dependent were observed (Figure 1c,d). *T. pseudonana* cells exhibited sustained growth at 12 °C with higher cell density, and a short-time rapid growth at 25 °C with lower cell density regardless of P condition. At 12 °C, the cell density almost showed no change during the first 24 h and then increased steadily in the 2-AEP and DIP groups until D6, sharing no difference in the first 4 days (Figure 1c). When cultured at 25 °C, the cell density exhibited a rapid growth in the first 48 h and then entered the stationary phase after D4 in the DIP group. The cells ceased growth after D3 in the 2-AEP group and then decreased significantly. Consistently, the growth rate of *T. pseudonana* is higher under 8 °C~17 °C than that under 17 °C~25 °C with sufficient DIP supply [29]. 

A significant difference was found in the promotion effect under different P conditions (Figure 1c). In the DIP-replete groups, the final cell concentration at 12 °C was slightly higher than at 25 °C. Meanwhile, in the 2-AEP groups, the final cell concentration in the 12 °C culture was about three times higher than that in the 25 °C culture. When cultured at 12 °C, the highest growth rate on D6 in the 2-AEP group was 0.152 ± 0.049 μ d^−1^, which is 66.4% of that in the DIP-replete group (0.228 ± 0.034 μ d^−1^, Figure 1c). 

Variations of Fv/Fm showed a comparable pattern in accordance with the growth curve (Figure 1d). The dramatic increase in Fv/Fm values in the first 24 h indicated that instant recovery of photosynthetic capacity accounts for the rapid growth in both cultures under 25 °C. After day 3, the Fv/Fm values declined continuously along with the cells entering the stationary phase in 25 °C cultures. In contrast, the Fv/Fm values increased steadily to meet sustained cell growth under 12 °C, and they were higher in the DIP-replete group than in the 2-AEP group. 

These results showed that *T. pseudonana* cells cultured with 2-AEP exhibited better physiological adaptability under lower temperatures, resulting in significantly increased cell density. Meanwhile, the Fv/Fm value represents a good consensus of higher photosynthetic capacity and a higher growth rate. 

### 3.3. Changes in Cellular Elemental Stoichiometry of T. pseudonana 

Many studies have revealed that marine phytoplankton elemental stoichiometric ratios deviate from the empirical Redfield ratio of 106C:16N:1P [30], thus playing a major role in shaping the environmental stoichiometry ratio [31]. 

#### 3.3.1. Stoichiometry Variation under Different P Conditions

**Figure 2:**
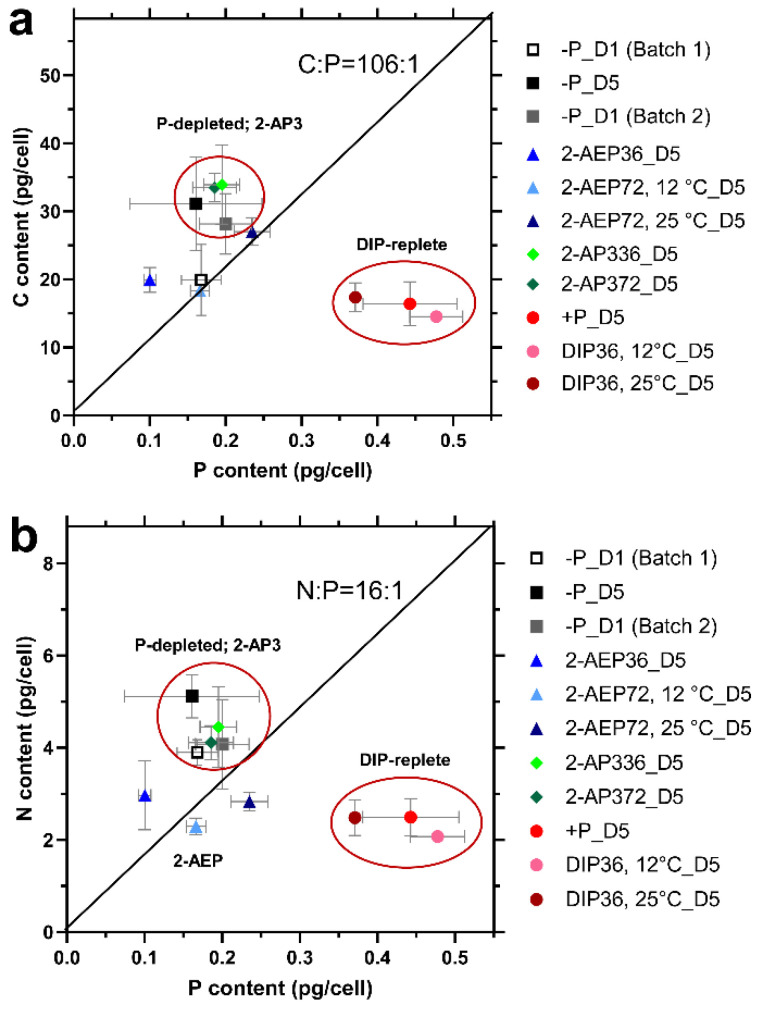
Cellular stoichiometry of *T. pseudonana* under different conditions. (-P, 2-AEP36, 2-AEP72, 2-AP336, and 2-AP372 represent P-depleted, 36 μM 2-AEP, 72 μM 2-AEP, 36 μM 2-AP3, and 72 μM 2-AP3 groups, respectively. D1 and D5 represent the first day and fifth day of the cultural period, respectively). (**a**) C:P ratio; (**b**) N:P ratio. Each culture group was set up in biological triplicate. The error bar represents the standard deviation of the mean values.

In this study, the N:P ratio of the DIP-replete group (~6:1) (Figure 2b) was far below that of the Redfield ratio and the group-specific optimal value of 14:1 [32]. This finding can be explained by the luxury uptake of DIP and storage in the form of polyP after P starvation, which is typical in diatoms [33,34]. Regarding the initial P starvation state, the C:P and N:P ratios were 117.72 ± 23.14 and 24.25 ± 7.94, respectively, consistent with reported cellular stoichiometry in diatoms under insufficient nutrient conditions [35]. Afterwards, the C:P and N:P ratios decreased significantly to 36.93 ± 3.96 and 5.63 ± 0.56, respectively, at D5 in the DIP-replete group, whereas they barely changed or increased under other different P conditions (Figure S2a). 

The stoichiometry of 2-AP3 groups (36 μM and 72 μM) was similar to that of the P-depleted group (Figure 2), in line with the growth pattern. The P-depleted cells and those treated with 2-AP3 were grouped together, showing higher C:P and N:P ratios, mainly because of higher cellular C and N contents and lower cell P content than that in the DIP-replete groups (Figure S3a).

#### 3.3.2. Effect of Temperature on Cellular Stoichiometry

In the DIP-replete groups, no significant difference was identified between 12 °C and 25 °C (Figure 2 and Figure S2b). In the 2-AEP group, the C:P and N:P ratios were lower than those in P-depleted *T. pseudonana* and higher than those in the DIP-replete group. In the 72 μM 2-AEP cultures (12 °C and 25 °C), the C:P and N:P values were 111.2 ± 11.1~115.8 ± 12.1 and 13.9 ± 1.8~12.1 ± 0.6, respectively, closer to the Redfield ratio with higher 2-AEP concentration and lower temperature.

The interactions between environmental conditions and cell growth are the key factors driving stoichiometric variation [36]. Temperature is the major factor due to its direct effect on cell growth [34,37]. Global research on phytoplankton stoichiometry has found that C:P and N:P ratios decrease with temperature [37,38,39], but laboratory evidence regarding species differences is insufficient. The findings of the present study show the synergetic effect of temperature and phosphonates on cellular stoichiometry in *T. pseudonana*, higher N:P ratios when cultured with 2-AEP under lower temperature. In the DIP-replete group, the lower temperature significantly decreased the C:P and N:P ratios of *T. pseudonana*, consistent with previous reports [37,40,41].

Cells increase ribosome concentration and cellular P content to compensate for low translation efficiency of ribosomes at low temperature [42]. Such a hypothesis is consistent with the observation of the strong temperature dependency of C:P and N:P in high-latitude ecosystems [43]. In this study, the cellular P content of the DIP-replete groups was significantly higher at 12 °C than at 25 °C (Figure S3b), indicating that low temperature may increase the P demand and promote the P absorption of *T. pseudonana*. In addition, temperature had a significant effect on the C:P and N:P ratios in the DIP group. In the 2-AEP group, temperature effects were barely observed on the stoichiometry of *T. pseudonana*. This finding may be attributed to the stable chemical properties of 2-AEP, which is hard to be hydrolyzed into phosphate during cellular P determination.

### 3.4. High Variability of Stoichiometry in Diatom

**Figure 3:**
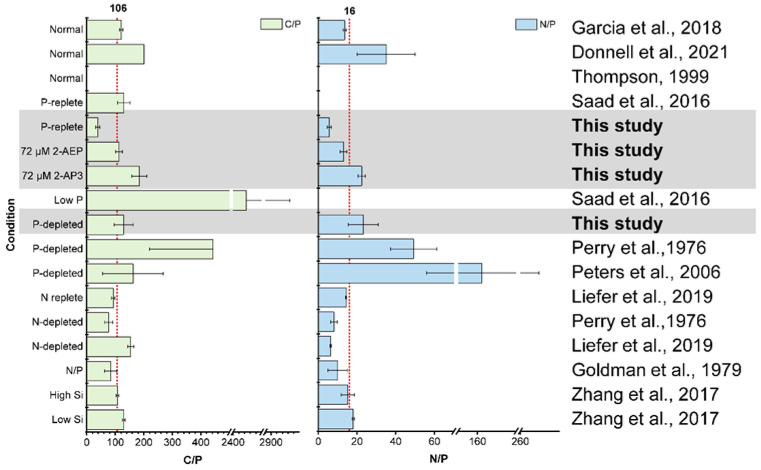
Comparison between the elemental stoichiometry of *T. pseudonana* in this study and that in previous research. Normal represents the nutrients in adequate culture conditions, P-replete represents DIP resupplied to the phosphorus-starvation cells, P-depleted represents the phosphorus-starvation condition, and low P represents a very low phosphorus concentration in the medium [37,45,46,47,48,49,51,52,53].

Nutrient availability is considered to be the major driver shaping phytoplankton stoichiometry; for example, P limitation accounts for the increase in C:P and N:P ratios of phytoplankton [44,45]. In this research, the C:P and N:P ratios of *T. pseudonana* declined rapidly after 36 μM DIP supplementation. The C:P and N:P in this study were much lower under the same or similar P conditions than those in other studies (Figure 3) [37,45,46,47]. In agreement with the previous results, the C:P and N:P ratios of DIP-depleted *T. pseudonana* were higher than the Redfield ratio [45,48,49]. According to the observation data from ALOHA and BATS stations, the C:P ratio of suspended particles varies mostly from 100 to 200 and below the P-stressed threshold [50]. Our results show that *T*. *pseudonana* grown with 2-AEP is close to the classical Redfield ratio.

The effects of other nutrients, such as N or Si, have been studied. N deficiency leads to a decreased N:P ratio [48,51,52], and Si concentration has no significant effect on both C:P and N:P ratios (Figure 3) [53]. Through summarizing and comparing with previous reports, our study provides fundamental information for addressing temperature and P nutrient effects on stoichiometry variation in *T. pseudonana*.

## 4. Conclusions

Overall, this study has three major findings. (1) Biogenic phosphonates 2-AEP and 2-AP3 can be utilized by diatom *T. pseudonana* to support cell growth, and 2-AEP is more preferable than 2-AP3, as evidenced by higher cell density. (2) Disparate growth strategies are identified under different temperatures, and the significantly promoted cell growth of the 2-AEP culture under lower temperature than mild temperature indicates its adaptive function. (3) The mediate value of C:P and N:P ratios in the 2-AEP groups between that in P-depleted (2-AP3) and DIP-replete groups suggests the potential effect on environmental elemental stoichiometry.

The results of this study provide new insights into interpreting the alternative P nutrient strategy adopted by diatoms in different scenarios. In sub-polar regions, diatoms represent the major primary producers and may benefit from taking 2-AEP to support growth. Another bold hypothetical scenario is in inorganic nutrient-scarce coral reef ecosystems, where diatoms may obtain advantages by using biogenic phosphonates released by metazoans [54], which require further study. The findings of stoichiometry variation of *T. pseudonana* under different P and temperature conditions provide further understanding of the diatom ecophysiology in marine biogeochemical cycling.

## Figures and Tables

**Table 1:** Culture conditions (a, b: control groups in batch 1, b: control group in batch 2).

Culture	T (°C)	P Nutrient Concentration	Culture Condition
Seed		Starvation treated 8–10 days (<0.3 μM)	f/2 medium (salinity = 30) 14:10 light: dark cycle photon flux: 180 μmol m^−2^ s^−1^ Antibiotics cocktail (final concentration in medium: 100 mg L^−1^ ampicillin, 50 mg L^−1^ streptomycin, and 50 mg L^−1^ kanamycin)
−P ^a^		No addition	
+P ^b^		DIP (36 μM)	
Batch 1	20	2-AEP (36 μM)	
		2-AP3 (36, 72 μM)	
Batch 2	12/25	2-AEP (72 μM)	

## Data Availability

Data are contained within the article and Supplementary Materials. They are available on request from the authors.

## Supplementary Materials

The following supporting information can be downloaded at: https://www.mdpi.com/article/10.3390/microorganisms12040761/s1, Figure S1: Growth curve of *P. tricornutum* under 2-AP3 treatment. Figure S2: Stoichiometry of *T. pseudonana* under different phosphorus and temperature conditions. Figure S3: Variations of cellular C, N, and P of *T. pseudonana* under different phosphorus and temperature conditions.
