# Supplementary material for: Biogenic Phosphonate Utilization by Globally Distributed Diatom Thalassiosira pseudonana
Source: Microorganisms. 2024 Apr 10;12(4):761. doi: 10.3390/microorganisms12040761 (PMC11051927; doi:10.3390/microorganisms12040761)
Supplement: Supplementary file 1 [file microorganisms-12-00761-s001.zip › microorganisms-2915945-supplementary.pdf]

Supplementary materials

Biogenic Phosphonate Utilization by Globally Distributed Diatom *Thalassiosira pseudonana*

Huilin Shu [1,2,4], Yuan Shen [1,3], Hongwei Wang [1,3], Xueqiong Sun [1,3], Jian Ma [1,2] and Xin Lin [1,3,*]

[1] State Key Laboratory of Marine Environmental Science, Xiamen University, Xiamen 361005, China; shuhuilin@jxnu.edu.cn (H.S.); yuanshen@xmu.edu.cn (Y.S.); wanghongwei@stu.xmu.edu.cn (H.W.); 22320220156409@stu.xmu.edu.cn (X.S.); jma@xmu.edu.cn (J.M.)
[2] College of the Environment and Ecology, Xiamen University, Xiamen 361005, China
[3] College of Ocean and Earth Sciences, Xiamen University, Xiamen 361005, China
[4] College of Life Sciences, Jiangxi Normal University, Nanchang 330022, China
* Correspondence: xinlin@xmu.edu.cn

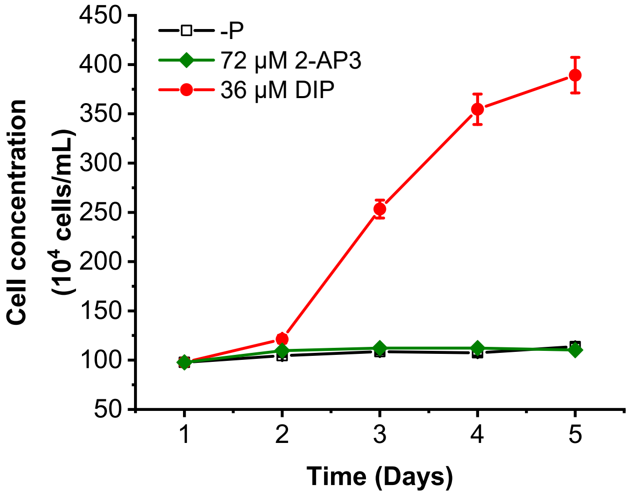

Figure S1. Growth curve of *P. tricornutum* under 2-AP3 treatment.

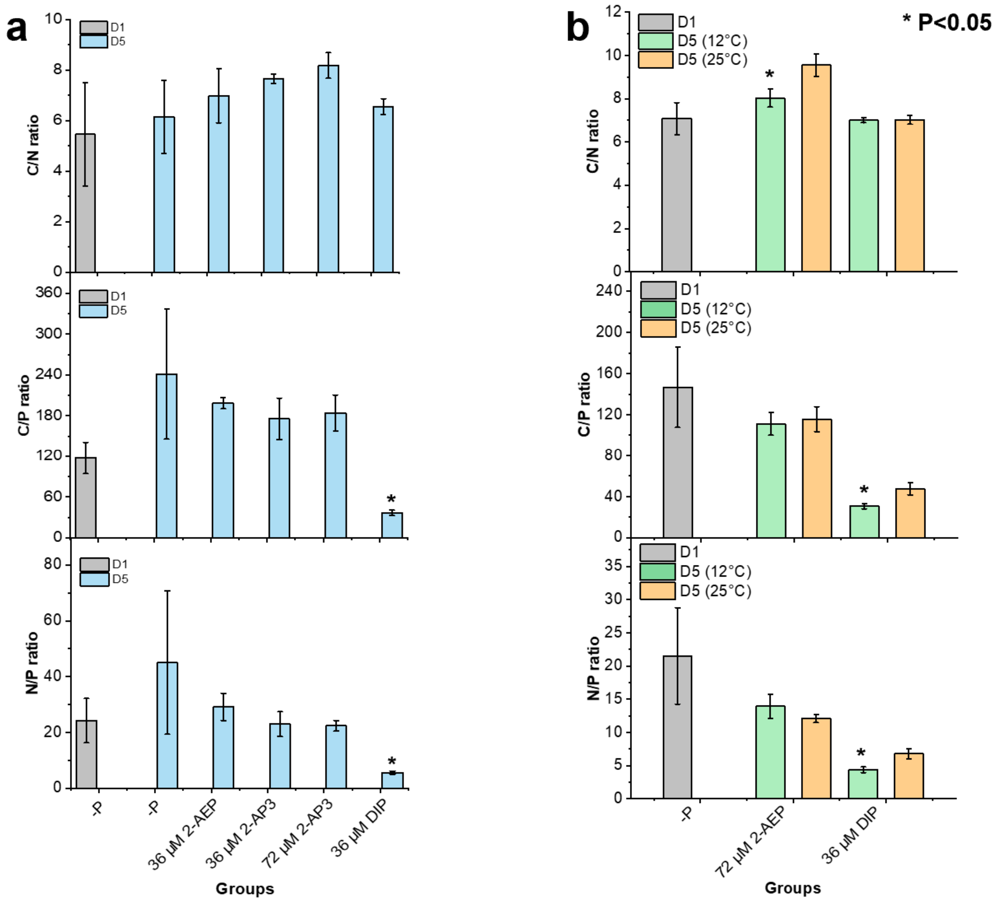

Figure S2. Stoichiometry of *T. pseudonana* under different phosphorus (**a**) and temperature (**b**) conditions. * represent the significant difference between (**a**) P-depleted and other phosphorus treated group, and (**b**) between 12 °C and 25 °C.

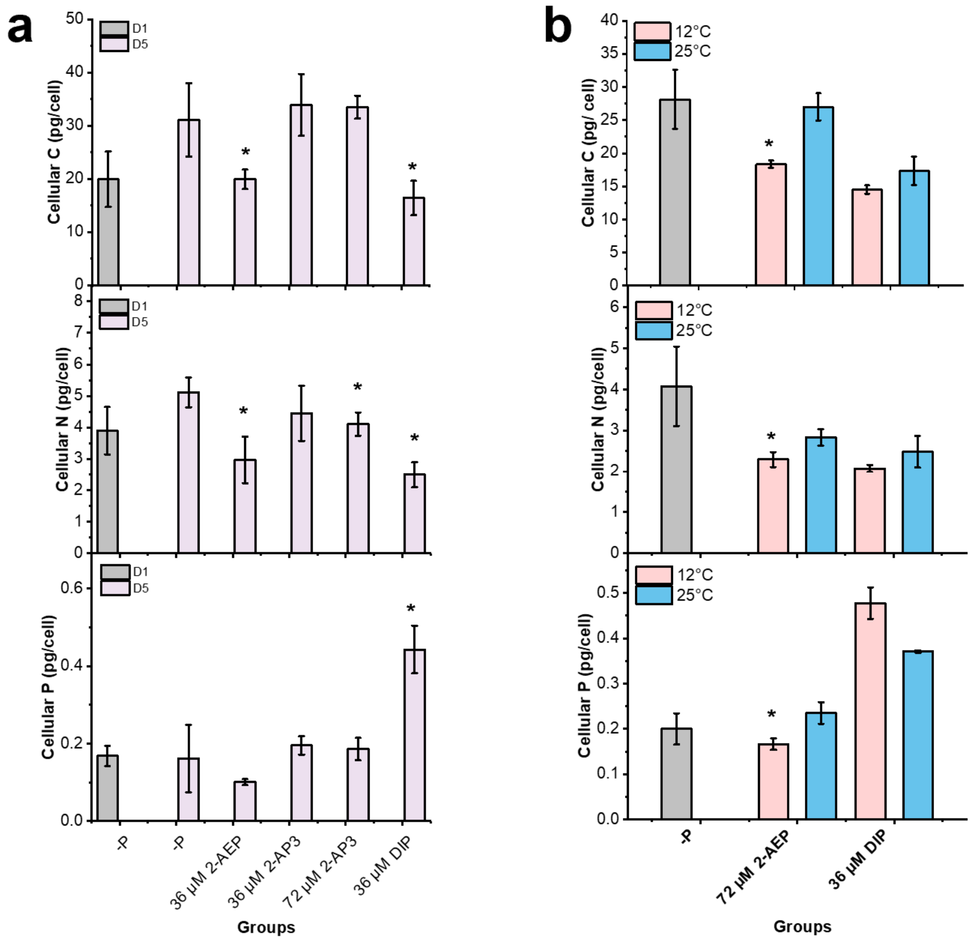

Figure S3. Variations of cellular C, N and P under different phosphorus (**a**) and temperature (**b**) conditions. * represent the significant difference (p <0.05) between (**a**) P-depleted and other phosphorus treated group, and (**b**) between 12 °C and 25 °C.
